# COVID-19 pandemic and antimicrobial resistance: Another call to strengthen laboratory diagnostic capacity in Africa

**DOI:** 10.4102/ajlm.v9i1.1302

**Published:** 2020-09-23

**Authors:** Beverly Egyir, Noah Obeng-Nkrumah, George B. Kyei

**Affiliations:** 1 Bacteriology Department, Noguchi Memorial Institute for Medical Research, University of Ghana, Accra, Ghana; 2 Department of Medical Laboratory Sciences, University of Ghana, Accra, Ghana; 3 Virology Department, Noguchi Memorial Institute for Medical Research, University of Ghana, Accra, Ghana

## Introduction

The coronavirus disease 2019 (COVID-19) pandemic caused by the severe acute respiratory syndrome coronavirus 2 (SARS-CoV-2) resulted in 7 145 539 confirmed cases and 408 025 deaths by 03 June 2020.^1^ Understandably, almost all attention has been on COVID-19, which continues to take lives and stretch healthcare systems around the world. A critical issue receiving much less attention during this pandemic is the effect it could have on antimicrobial resistance (AMR). With no proven therapy for COVID-19, prescribers are more likely to use antibiotics indiscriminately for treatment and prevention of presumed bacterial co-infections. In high-income settings, where bacterial cultures can be done on a timely basis, such antibiotic regimens can be stopped within 48 hours. However, in Africa, unneeded empiric antibiotic regimens are likely to be continued for a longer period of time due to lack of bacterial culture and antimicrobial susceptibility testing (AST) capabilities. In addition, the widespread use of hand sanitizers and antimicrobial soaps could exacerbate AMR among healthcare workers and the general population. In this article, we discuss how the COVID-19 pandemic could affect the already precarious AMR situation in Africa and discuss ways to minimise the effects on public health.

## Antimicrobial resistance, a global menace

Antimicrobial resistance occurs when microbes change and render antimicrobial agents ineffective. This phenomenon is fuelled by overuse and misuse of antimicrobial agents in humans and animals. Antimicrobial resistance leads to treatment failures, prolongs hospital stays, worsens clinical outcomes and makes surgical procedures and chemotherapy risky and unsafe. With increasing antibiotic resistance and few new antimicrobial agents in the production pipeline, it is imperative to monitor the epidemiology of bacteria species, especially respiratory pathogens to inform treatment decisions^2,3^ in the era of COVID-19. It has been estimated that AMR could lead to 10 million deaths by 2050 if nothing is done about the menace, and most of the deaths are likely to occur in Asia and Africa.^4^ Actions such as infection prevention and control, antibiotic stewardships, capacity building (laboratory infrastructure and personnel) and surveillance are necessary to ameliorate the impact of AMR. To support a global action plan on AMR, the World Health Organization has developed the Global Antimicrobial Resistance Surveillance System to provide a standardised approach for collection, analysis and sharing of data related to AMR to inform decision-making at national and international levels; surveillance of AMR bacteria in humans and animals across the globe is key in tackling the problem of AMR.^5^ Unfortunately, this is hampered by the limited data from Africa due to limited diagnostic microbiology infrastructure on the continent.^6^

## Coronavirus disease 2019, secondary bacterial infection and antimicrobial resistance

Viral respiratory infections can be complicated with secondary bacterial infections, commonly with *Streptococcus pneumoniae, Haemophilus influenzae* and *Staphylococcus aureus*, resulting in increased severity and mortality.^7^ The majority of deaths from the 1918 influenza pandemic resulted from secondary bacterial infections.^8^ During the 2009 swine influenza pandemic, there was an increase in hospital pneumonia cases as a result of secondary bacterial pneumonia, which resulted in 29% – 55% of deaths.^9^ In a recent study on the outcome of COVID-19 patients from the Wuhan region of China, half of the non-survivors had secondary infections.^10^ Of note, the majority (95%) of the patients in the study were given antibiotics.^10^

Azithromycin, a broad-spectrum macrolide antibiotic (in combination with hydroxychloroquine) has become a common treatment for COVID-19 patients in several parts of the world including Africa^11,12^ without much evidence to support its use. It is worth noting that the World Health Organization has warned against indiscriminate use of antibiotics during COVID-19 treatment.^2^

While antibiotics may not affect COVID-19 directly, they are agents administered to prevent or treat secondary bacterial infections in COVID-19 patients. Therefore, the swell in the numbers of COVID-19 patients could lead to the inappropriate use of antibiotics and subsequent selection of AMR bacteria. In previous epidemics, there were indications of an increase in methicillin resistant *S. aureus* infections in hospitals, which was linked to heavy use of antimicrobial agents.^13,14^ It is therefore possible that COVID-19 patients could also be battling with AMR bacteria, in addition to the virus, resulting in poor patient outcomes. Urgent studies are needed to define the bacteria aetiologies and AMR bacteria that contribute most to mortality in COVID-19 patients.

## Coronavirus disease 2019, the environment and antimicrobial resistance

From the onset of the pandemic, the general public has been constantly directed to regularly wash hands with soap and water, and use hand sanitizers. Although such practices will help improve hygiene standards and reduce the spread of COVID-19, they could have negative effects on AMR. Disinfectants and antimicrobial soaps contain biocides or antimicrobials. An increase in use of these agents may lead to an increase in their concentration in waste water and receiving water bodies; this may have a potentially negative impact, because the elevated concentrations may lead to selection of AMR bacteria, posing a health risk to persons exposed to such environments.^15^ More research in Africa is needed to determine how the widespread use of antimicrobial sanitizers and soaps affects the microbiome and contributes to AMR.

## Antimicrobial resistance in Africa in the era of coronavirus disease 2019

The World Health Organization reports that there is limited data on AMR prevalence from Africa due to limited laboratory capacity and surveillance networks.^6,16^ A survey indicated that very few countries in Africa have functional national surveillance systems for AMR of common bacterial infections from community and hospitalised patients.^17^ An external quality assessment report indicated poor performance of AST in several African countries.^18^ Quality assurance in AST is key for reporting and implementation of the Global Antimicrobial Resistance Surveillance System^19^; laboratories in Africa have to be supported with the needed investments for better performance.

With the paucity of data on AMR, realistic evidence on how AMR may compromise first-line empirical treatment in common bacterial infections is lacking on the continent.^16^ In the absence of microbiology and AMR data, prescribers often manage clinical symptoms rather than specific bacteria,^16^ a situation that may fuel inappropriate use of antimicrobials and emergence of resistant microbes.

In Ghana, like most African countries, the majority of microbiology laboratories do not have the capacity to perform culture and AST tests.^20^ For the few laboratories that are able to do culture and AST, performing such tests in a standard way is a challenge often due to a lack of reference strains and up-to-date standard interpretation guidelines. In addition, culture of bacteria and AST are often not requested by clinicians, because of cost to patients and the long turn-around time that often render the results useless for patient care. Altogether, data on AMR bacteria to guide treatment decisions at local and national levels are scarce. Newman et al.^20^ conducted the first nationwide AMR surveillance in Ghana between 2002 and 2003 and found bacteria species resistant to ceftriaxone (6.3%) and ciprofloxacin (11%).^21^ Opintan et al. (2015), followed up with another nationwide survey between June 2014 and November 2014 and observed that > 50% of the commonly isolated bacteria species were resistant to third-generation cephalosporins and fluoroquinolones. In this study, the majority of the Gram-negative bacteria species recovered were positive for extended spectrum beta lactamase; these organisms are resistant to a wide range of antimicrobial agents.^22^

In another study, a New Delhi metallo-beta-lactamase-producing *Escherichia coli* strain resistant to meropenem and belonging to ST410 was detected in a urine sample from a hospitalised patient in the northern part of Ghana. In this first report, the detected plasmid co-carried other resistance genes.^23^ This is disturbing, mainly because these organisms are resistant to the antimicrobials used as last-line treatment for severe bacterial infections.^24^

Among female patients who presented with vaginal discharge, dysuria, intermenstrual bleeding or abdominal pain and men who presented with urethral discharge or dysuria, gonococcal isolates recovered from samples collected from five healthcare centres were resistant to tetracycline (100%), benzylpenicillin (91%) and ciprofloxacin (82%). One isolate resistant to cefixime (MIC: 0.75 *µ*g/ml) belonged to ST1407, a pandemic resistant clone.^25^

A total of 520 methicillin susceptible *S. aureus* and 30 methicillin resistant *S. aureus* isolates were recovered from 1219 nasal swabs (community and hospital carriers) and 916 clinical isolates (blood, skin and soft tissues, wounds) in other studies; methicillin resistant *S. aureus* isolates detected belonged to global epidemic clones, including USA300.^26,27,28,29^

Our review of more than 20 articles on AMR from Ghana for this write-up revealed that resistance of bacteria species (recovered from human, food and animals) to commonly used antimicrobial agents such as ampicillin, tetracycline, chloramphenicol and trimethoprim sulfamethoxazole was common. The situation is not different from other parts of the continent.^16^ The majority of AMR data in Ghana are from pockets of studies focusing on particular bacteria species recovered in select hospitals or research institutions. They therefore may not reflect the national AMR situation and could be an underestimation of the actual magnitude of the AMR problem in a country where self-medication is rampant and antimicrobial agents are often available without prescription.^30^

Ghana is gradually gathering momentum to get to a stage of having a functional national system to monitor AMR. The Antimicrobial Use and Resistance Policy and the national action plan on AMR, which provide strategies and plans to guide AMR data generation for evidence-based interventions at local, national and international levels, were launched by the President of Ghana on 11 April 2018. The national action plan was fashioned along the lines of the objectives of the Global Action Plan on AMR.^5^ Ghana also has a national AMR working group; the group meets regularly to deliberate on AMR issues in the country. To strengthen knowledge and evidence base through surveillance and research, Ghana received the first Fleming Country Grant, which is currently supporting a government-led system of collecting, analysing and reporting of AMR and antimicrobial use data from humans and animals from 11 sentinel sites. These data will provide a national AMR picture to inform treatment decisions at the national level. Plans are underway to begin a pilot surveillance study.

The prevalence and mortality rate of COVID-19 in Africa is lower compared to that of Europe, America and Asia. However, the rapid spread of the virus is another call to strengthen the laboratory diagnostic capacity in Africa to perform standard antimicrobial susceptibility testing, especially of relevant respiratory pathogens to inform treatment decisions in healthcare facilities. Importantly, AST data need to be collected yearly to support empiric treatment in hospitals. Continuous surveillance is required on the African continent and across the globe to understand the epidemiology of AMR pathogens, provide the needed data to guide and inform treatment decisions and policies and monitor resistance trends and emergence of new clones. In all of these actions, the role of networks and collaborations in enhancing the success of various interventions cannot be overemphasised. Ownership and sustainability plans by governments and local authorities are key to maintaining the successes for continuous AMR surveillance activities with local and international partners. Capacity building (infrastructure and personnel) is a must-have and must be continuously improved to fight AMR in Ghana and Africa.

## Going forward

One cannot blame the African prescribers for throwing the ‘kitchen sink’ of antibiotics at sick and dying COVID-19 or other virally infected patients. Rather, these practices could be reduced significantly, if physicians were empowered to order cultures and obtain results in a timely manner. Doctors faced with negative culture results are more likely to stop antibiotics than if no results are available. With the necessary political will, African governments and academics can do a few things in line with the five strategic objectives of the Global Action Plan on combating AMR during the current pandemic and beyond. Firstly, there is a need to raise awareness of AMR among personnel in human and animal health, and agriculture, as well as among consumers, to ensure a proper understanding of AMR pathogens and the impact of AMR across sectors. There is an urgent need to build laboratory capacity to generate the required microbiology data through surveillance and research to inform treatment decisions, especially in urban centres where resistant organisms are often abundant. Evidence-based prescribing and dispensing should be the way to go to optimise antimicrobial use. More automated systems like the GeneXpert platform used for tuberculosis could be repurposed for organisms like methicillin-resistant *S. aureus*. In addition to phenotypic methods used in the detection of resistance, genomic tools such as whole genome sequencing can be utilised to generate extensive data to expand our knowledge on the changing epidemiology of AMR bacteria. During the COVID-19 pandemic, urgent studies are needed to document the bacterial organisms responsible for co-infections at the local level to guide empiric therapy. Due to the widespread use of hand sanitizers and antimicrobial soaps, research is needed to study healthcare workers and others for changes in the skin flora that they carry, which could be transmitted to vulnerable patients. Healthcare institutions without antibiotic stewardship programmes must take steps to institute measures for rational antibiotic use. Antibiotic stewardship optimises institutional antibiotic use and reduces the selection and spread of AMR; similarly, infection prevention needs strengthening across the board to limit the spread of resistant bacteria. Finally, the need for increased investment by African governments to drive development of new diagnostic tools, novel antimicrobial agents and vaccines cannot be over-emphasised.

## Lessons and conclusion

The AMR menace has been described as a problem that knows no borders; resistant bacteria can be found in humans, animals, food and the environment. The current pandemic shows that we remain susceptible to infections for which we have no therapeutic options. The experience from COVID-19 therefore should be another reminder of the life-threatening consequences of AMR microbes, and the need for rapid capacity building (infrastructure and human resources) for surveillance of resistant bugs on the African continent.
